# Comparative Risk of Bleeding of Anticoagulant Therapy with Vitamin K Antagonists (VKAs) and with Non-Vitamin K Antagonists in Patients Undergoing Dental Surgery

**DOI:** 10.3390/jcm10235526

**Published:** 2021-11-25

**Authors:** Mattia Manfredini, Pier Paolo Poli, Luca Creminelli, Alberto Porro, Carlo Maiorana, Mario Beretta

**Affiliations:** Fondazione IRCCS Ca’ Granda Ospedale Maggiore Policlinico, Implant Center for Edentulism and Jawbone Atrophies, Maxillofacial Surgery and Odontostomatology Unit, University of Milan, Via della Commenda 10, 20122 Milan, Italy; mattiamanfredinidr@gmail.com (M.M.); PierPaolo.Poli@unimi.it (P.P.P.); luca.creminelli@unimi.it (L.C.); carlo.maiorana@unimi.it (C.M.); Mario.Beretta@unimi.it (M.B.)

**Keywords:** oral anticoagulants, NOAC, oral surgery, dental extractions, dental surgery, hemostatic measures

## Abstract

Objectives: A wide variety of approaches have been proposed to manage anticoagulant drugs in patients undergoing dental surgery; vitamin K antagonists and novel direct oral anticoagulants have been used. The present study aims to explore the existing evidence concerning the management of patients in anticoagulant therapy undergoing oral surgery procedures and to give suggestions related to peri- and post-operative measures. Materials and methods: A comprehensive search of databases was conducted to identify studies that evaluated the relationship between direct oral anticoagulants and dental procedures. The present scoping review was realized in adherence with the Preferred Reporting Items for Systematic reviews and Meta-Analyses extension for Scoping Reviews (PRISMA-ScR) guidelines. The publications varied from randomized controlled trials (RCT) to cohort trials. Only articles written in English language and published between 2000 to 2020 were screened. The studies were included if discussing the management of a patient in anticoagulant therapy (warfarin or direct oral anticoagulants) scheduled for tooth extraction. Results: 33 studies were selected and included in the qualitative review. Nineteen considered anticoagulant therapy with warfarin, six considered anticoagulant therapy with new oral anticoagulants and eight compared patients taking warfarin with patients taking direct oral anticoagulants. Conclusions: No case of extractive surgery should alter the posology of the drug: thromboembolic risks derived from discontinuation are heavier than hemorrhagic risks. Clinical relevance: direct oral anticoagulants are safer in terms of bleeding and manageability and bleeding episodes are manageable with local hemostatic measures.

## 1. Introduction

As the average age of the population increases, more elderly patients need to be referred for oral surgery, even if it is a simple tooth extraction. These patients often have a medical history and ongoing drug therapy.

The two main types of anticoagulants are Vitamin K antagonists (VKAs) and the new oral anticoagulants (NOACs), known also as direct oral anticoagulants (DOACs). The most important VKAs are Warfarin and acenocoumarol. There are derivatives of 4-hydroxycoumarin. The targets of VKAs are factors VII, IX, X and proteins C, S and Z [1]. Warfarin therapy reduces the risk of arterial thromboembolic events such as strokes by 70% [2] and the risk of recurrent venous thromboembolism by 90% [3]. The anticoagulative effect develops after a latency of ~48 h and takes 4 to 5 days to be fully active with a half-life of at least 48 h [4]. This means that the anticoagulant effect persists even after discontinuation for a comparatively long time and that must be considered before planning any surgery [5]. To increase and accelerate the anticoagulant effect, the dosage can be raised to a maximum of 75 mg/kg, above which the effect will no longer be accelerated [6]. Prothrombin time (PT) is a blood test that quantifies the time it takes for a fibrin clot to form. PT is usually expressed by an index called INR (International Normalized Ratio). The INR value under normal conditions is 0.9/1.2, but the physician can establish optimal values tailored to the patient. Many guidelines describe the desirable value of the INR for patients taking oral anticoagulants, ranging between 2 and 3. Some studies have demonstrated that exodontias performed in patients with a recommendable INR range can be safely conducted without oral anticoagulation interruption or antiplatelet drugs [7,8]. Those recommendations include a reduction, a temporarily interruption of OAT (oral anticoagulant therapy) or a substitution (bridging) with heparin prior to surgical procedure [9,10,11].

The new oral anticoagulants, instead, act as selective inhibitors of factor II, X or platelet aggregation inhibitors). Differently from VKAs, DOACs have a more manageable posology and have no need for laboratory tests to monitor INR fluctuations. Selectively blocking the cascade factors of coagulation, these drugs impede the conversion of fibrinogen into fibrin strands, with an effective anticoagulant action [12].

These drugs represent a risk in oral surgery, especially in extractive surgery. The risks range from simple obstruction of the surgeon’s field of vision to the possibility of bleeding episodes due to anticoagulation. Bleeding episodes are also favored in extractive surgery because of second intention healing of the socket, and it is therefore necessary to supplement the procedure with effective local hemostatic measures. In patients treated with these molecules and scheduled for oral surgery procedures, scientific evidence for withdrawal, reduction or continuation of the drugs is limited. Kwak et. Al., in a retrospective study, remarked the importance of the type of surgery, finding that the discontinuation of anticoagulant therapy with DOACs (rivaroxaban, dabigatran, apixaban, edoxaban) should change depending on the type of surgery (implant insertion, tooth extractions); nonetheless, the bleeding risk was under control in 144 cases out of 153 [13]. In a recent systematic review, J. Chahine et al. evaluated five RCTs and five case–control studies. The population considered was formed by patients in therapy with VKAs and with NOACs, that required an oral surgery procedure, namely, tooth extraction or implant placement (the two most performed oral surgeries). The main conclusion was that VKAs must be continued in all surgical procedures if INR is in the therapeutic range. As for NOACs, they must also be maintained in most procedures. Local hemostatic agents are mostly needed in both cases [14]. However, out of 10 studies, only one included patients in therapy with NOACs. For such reason, additional evidence is needed to understand how to manage dental patients in therapy with NOACs from a pharmacological standpoint.

In view of the above, the present study aims to explore the existing evidence concerning the management of patients in anticoagulant therapy undergoing oral surgery procedures and to give suggestions related to peri- and post-operative measures. Studies were screened to compare different types of anticoagulant drugs.

## 2. Materials and Methods

### 2.1. Protocol

The present scoping review was realized in adherence with the Preferred Reporting Items for Systematic reviews and Meta-Analyses extension for Scoping Reviews (PRISMA-ScR) guidelines in order to map evidence on a topic and identify main concepts and knowledge gaps [15]. The protocol of the present scoping review was registered at the National Institute for Health Research PROSPERO, International Prospective Register of Systematic Reviews (https://www.crd.york.ac.uk/PROSPERO, accessed on 15 March 2021 with the registration ID: CRD42021235862.

An adaptation of the PICO (Population, Intervention, Comparison and Outcome) model was used to build a focused question, consisting of a PEO (Population, Exposure, and Outcome) framework, to determine the association between a particular exposure and the outcomes [16]. This approach has been developed to perform qualitative systematic reviews in healthcare interventions [17] including oral surgery procedures [18]. The focused question was: “in patients scheduled for tooth extraction, in therapy with anticoagulant drugs including coumarin derivates (warfarin) or new oral anticoagulants (dabigatran, rivaroxaban, edoxaban, apixaban), how frequent are the bleeding events and which is the most adequate posology of the drug in terms of suspension or continuation, in the days approaching the surgery?”. The primary outcome was the occurrence of bleeding events, while the secondary outcome was the evaluation of other post-operative complications.

### 2.2. Eligibility Criteria

#### 2.2.1. Inclusion Criteria

To be included, every source of evidence had to fulfil specific inclusion criteria. Only articles written in English language and published between 2000 to 2020 were screened. Studies included in the screening process were randomized controlled trials (RCTs), controlled clinical trials (CCTs), retrospective and prospective case–control studies. No restrictions were placed on population characteristics, number of patients, age or systemic conditions. The studies were included if discussing the management of a patient in anticoagulant therapy (warfarin or DOACs) scheduled for tooth extraction (some studies considered other types of surgery), including the pre-operative pharmacological protocol, the post-operative measures, the number and the risk of bleeding events.

#### 2.2.2. Exclusion Criteria

All studies not fulfilling the inclusion criteria were excluded, such as (1) articles written in a language other than English; (2) case series, case reports, literature reviews; (3) studies that did not report neither the pharmacological management nor the evaluation of the bleeding risk; and (4) studies that did not add enough relevant data related to the topic.

### 2.3. Sources of Evidence

An electronic literature search was conducted in PubMed via MEDLINE, Scopus and Web of Science databases. The electronic search was complemented by a manual search of the reference lists of all selected full-text articles. The search aimed to collect relevant information about the perioperative measures and the changing in the posology in patients under anticoagulant therapy. In all databases, the years covered were from 2000 to 2020. The most recent research was executed on 10 January 2021.

### 2.4. Search Strategies

For all the libraries, a combination of specific keywords, medical subject headings [MeSH] and other terms not indexed as MeSH were used to identify all pertinent studies, according to the precise indications of the PEO question. As such, for the PubMed library, articles were selected using the following terms: (Anticoagulants[MeSH Terms]) AND (Surgery, Oral[MeSH Terms]). For the Scopus library, the terms used were dental AND surgery AND dabigatran AND rivaroxaban AND apixaban AND edoxaban AND warfarin. Finally, the terms used in the Web of Science library were TOPIC: (direct oral anticoagulants) AND TOPIC: (dental surgery) AND TOPIC: (warfarin). In each research string, some filters were applied, such as type of publication (randomized controlled study, retrospective study, controlled study) and year of publication (2000–2020).

### 2.5. Selection of Sources of Evidence

Two reviewers (A.P. and L.C.), working independently, completed the initial screening of titles and abstracts of all included papers. Full-text articles were assessed independently, and the selections compared between the two investigators. The final list and every disagreement between the two investigators were brought to the attention of a third and fourth investigator (M.M. and P.P.P.). Duplicate articles in the databases were identified and removed using EndNote Web reference manager software (Clarivate Analytics, Philadelphia, PA, USA). Some authors of the studies included were contacted to obtain unavailable full texts or to clarify uncertain and incomplete data.

### 2.6. Data Charting Process and Data Items

During the review process, an electronic spreadsheet (Microsoft Excel^®^, Redmond, WA, USA) was created and consecutively updated. All the useful information in each study was collected in tables. The demographic data were divided in categories: first author name, year of publication, title, type of study, database in which the study was found, number of patients participating, number of teeth extracted. Tables describing the primary and secondary outcome, the method of measurement of the primary outcome (bleeding events), the number and type of bleeding events were completed, along with tables containing all the data about the anticoagulant therapy including type of anticoagulant therapy (warfarin, DOACs, or both), suspension or continuation of the therapy, and other drugs taken by the patients. Additional tables were filled with information about comorbidities, time of post-operative follow-up, the eligibility criteria adopted in each study and the authors’ conclusions.

### 2.7. Methodological and Reporting Quality Assessment

Due to the specific research question herein, aiming to summarize the risk and the measure to consider in a patient taking oral anticoagulants and about to undergo a tooth extraction, no study quality assessment was performed. This is in accordance with the PRISMA-ScR guidelines stating that the risk of bias evaluation across studies is not applicable for scoping reviews.

### 2.8. Synthesis of Results

All data collection was done using a spreadsheet designed to express all the data regarding study characteristics and outcomes as tables in the results. As no meta-analysis was carried out due to the qualitative nature of the present scoping review and the impossibility to obtain an objective method of measurement of the primary outcome (bleeding events) evaluated in each study, a descriptive qualitative statistical approach was used to present the data.

## 3. Results

### 3.1. Selection of Sources of Evidence

**Figure 1:**
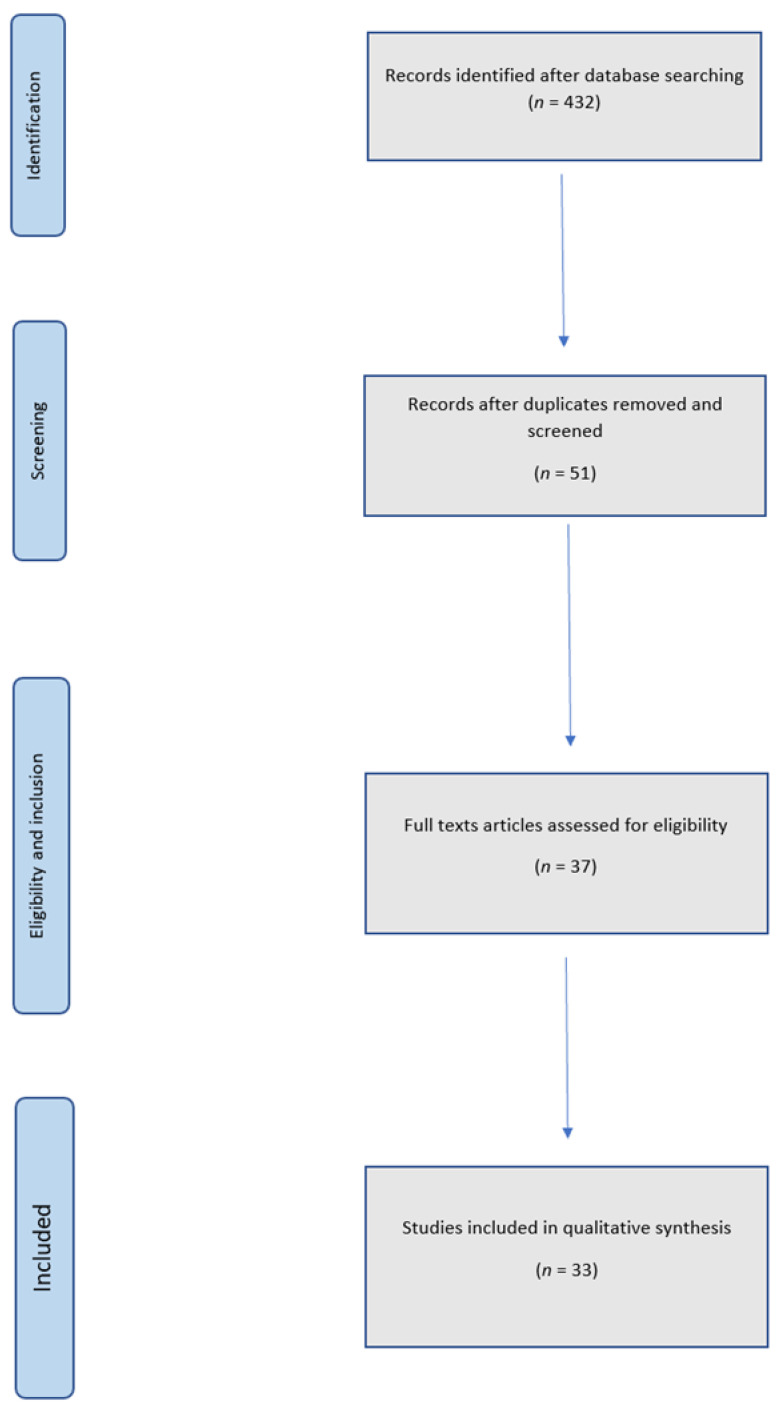
Flow chart of inclusion and selection.

Initially, 432 articles were found. Overall, 406 articles were identified in PubMed, nine in Scopus and 17 in Web of Science. Once all the duplicates were removed and the records were screened, 51 studies remained available. After applying the inclusion criteria, a total of 37 articles were identified. The final number of manuscripts included in the qualitative synthesis was 33. The flow diagram of the search and selection process is shown in Figure 1 and the studies were divided following the drug typology in Table 1, Table 2 and Table 3.

### 3.2. Characteristics of Sources of Evidence

Of the 33 studies included, 19 [4,19,20,21,22,23,24,25,26,27,28,29,30,31,32,33,34,35,36,37,38,39] considered patients in therapy with VKAs (warfarin and acenocoumarol) (Table 1).

A total of six studies [13,40,41,42,43,44,45,46] involved patients in therapy with NOACs (Table 2). In this category, four different types of drug were used for anticoagulation therapy: dabigatran, rivaroxaban, apixaban and edoxaban.

The remaining eight studies [47,48,49,50,51,52] were the most relevant to the aim of the present review, as they compared patients treated with VKAs with patients in therapy with NOACs (Table 3).

### 3.3. Results of Individual Sources of Evidence

#### 3.3.1. VKAs

Of the 19 studies including patients in therapy with VKAs, the discontinuation timing of the doses of the anticoagulant was not the same for each study. To overcome this drawback, two different groups were considered: 12 studies observed the occurrence of bleeding events when the therapy was not suspended, whereas seven studies considered at least one case/control group in which the therapy was suspended or bridged or modified.

1. Suspension of Anticoagulant

Sacco et al. [20] divided the patients in two groups. In group A, the INR was measured 72 h before the procedure. The dosages of acenocoumarol or warfarin were reduced until INR values between 1.5 and 2.0 were achieved on the day of the procedure, considering a target of 1.8. No local or general hemostatic measures were adopted during or after surgery. The INR was maintained at these levels for 24 h after surgery, and full anticoagulant dosage was resumed 48 h later. In group B, the INR was measured 72 h before the procedure and on the day of the surgery. Perioperative measures such as oxidized cellulose sponge, gelatin and tranexamic acid (TXA) mouthwash were used to control the bleeding. One-hundred-and-thirty-one patients were included: 66 in group A and 65 in group B. Statistically, there was no significant difference between the two groups at the end of the study: no immediate bleeding; 10 episodes of mild post-surgical bleeding in group A and six in group B; in each case, the bleeding was well handled by local measures. Cannon et al. [4] included a total of 70 patients in warfarin therapy, undergoing dental extractions. The first 35 patients were enrolled in the control group and had their warfarin treatment stopped for two days prior to the procedure. The INR level on the day of the procedure had to be lower than 2.0; otherwise, the surgery was postponed to another day. The subsequent 35 patients were included in the study group, with no discontinuation of therapy. In the control group, local hemostatic measures and sutures were used on every socket, while in the study group none of those measures were used. None of the patients, in either the control or the study group, had any immediate post-operative bleeding. However, intermittent oozing was recorded during the first 24 h period in three patients in the control group and in two patients in the study group. This was easily controlled with local pressure gauze. Statistically, there was no difference in the outcome between the two groups. Al-Mubarak et al. [23] divided 168 patients in therapy with warfarin in four groups: (1) no suture, warfarin not discontinued; (2) no suture, warfarin interrupted; (3) socket suture, warfarin continued; and (4) socket suture, warfarin interrupted. They observed that (1) there was no significance difference in terms of bleeding events in the four groups, and (2) the INR levels dropped to 1.5 in the two groups with discontinuation of therapy. Furthermore, the bleeding events were more frequent in the two groups involving the socket sutured. Sammartino et al. [27] included 84 patients divided in two groups made by 31 (control group) and 53 (study group) subjects each. The control group stopped the anticoagulation regimen a few days before surgery, obtaining an INR < 2. The study group maintained the regimen unchanged, achieving the control of hemostasis in the residual socket with absorbable swelling sponges loaded with TXA. There were only six bleeding episodes, and no significant difference between the groups. All the complications were handled successfully with a gauze moistened with TXA. Karsli et al. [28] formed four groups: continuation of warfarin, warfarin bridged with low-molecular-weight heparin (LMWH), warfarin bridged with unfractionated heparin and a control group of healthy individuals. Gauze weights used before and after blotting were measured with an electronic fine weight measurement device, and differences in weight before and after gauze blotting were interpreted as the amount of bleeding. No severe postoperative bleeding was detected in any patient, and the number of extra gauze swabs did not differ significantly among groups. Bajkin et al. [30] also studied the LMWH bridging approach in 105 patients compared to 109 subjects who continued with warfarin. In the latter group, warfarin was discontinued 3 to 4 days before the appointment, obtaining an INR level < 1.5 the day of the intervention. In cases of INR above this level, the surgery was delayed. LMWH bridging started one day after the interruption of warfarin and stopped 12 h before surgery. All the few cases of bleeding were easily solved with local hemostatic measures, and no statistically significant difference was found between these two groups of patients. Already in 2002, Evans et al. [34] started to reconsider the discontinuation of warfarin in patients undergoing simple dental extractions, stating that the risk derived by the discontinuation could be much worse than bleeding events caused by the continued regimen which can be handled well by local measures.

2. Continuation of Anticoagulant

The focus of the studies reviewed in this section is the use of local hemostatic measures in patients that did not discontinue anticoagulant therapy. Exceptions are the papers of Rocha et al. [37] and Bajkin et al. [29] in which hemostatic measures were not evaluated.

Rocha et al. evaluated the pattern of bleeding in individuals under continued VKAs therapy and non-anticoagulated individuals undergoing dental extractions. Perioperative bleeding was quantified through the storage of the fluids aspirated during the surgical procedure using a portable vacuum pump to achieve a final score for bleeding. The values varied from 2 to 14, with lower scores indicating less perioperative bleeding. The few bleeding events showed no statistical difference between the two groups.

Bajkin et al. conducted a study assessing the effect of combined oral anticoagulant–aspirin therapy on postoperative bleeding in patients undergoing tooth extractions. The three groups considered were (1) combined anticoagulant-aspirin therapy, (2) oral anticoagulant therapy and (3) aspirin therapy. Three cases of bleeding occurred in group 1, two cases in group 2 and no cases in group 3. All the events were easily solved by standard local measures.

Queiroz et al. [21] tried to provide the clinician with an effective method to control bleeding. They formed two groups: (a) socket compression with gauze moistened with saline solution and (b) socket compression with gauze moistened with TXA. The latter measure of local hemostasis in topical form with gauze compression and irrigation was shown to be more effective in reducing the time to attain immediate hemostasis and in preventing intermediate bleeding. Even before this, Al-Belasy et al. [24] studied another method of hemostasis control: the n-butyl-2-cyanoacrylate glue. The control group, not using such local measure, manifested a statistically significant number of bleeding events, while the study group showed reassuring results.

Da Silva et al. [25] in 2018 assessed a hemostatic agent that was not well documented in literature: epsilon-aminocaproic acid (EACA). EACA acts at the same level of the coagulation cascade as the TXA but is considered to be less efficient. The group formed with patients using EACA as local measure presented the same occurrence of late bleeding events as the control group using routine measures to control bleeding. Soares et al. [26] analyzed three groups: (1) gauze pad soaked in TXA and applied to the surgical alveolus for 8 min under biting pressure; (2) fibrin sponge packed into the surgical alveolus, biting down on dry gauze for 8 min for compression; and (3) dry gauze compression performed under biting pressure on the surgical alveolus for 8 min without the use of local hemostatic agents. There was no statistical difference between the three groups and between the three hemostatic measures, in terms of bleeding events. Scarano et al. [31] evaluated the use of calcium sulfate (CaS) as hemostatic agent. In group 1, the post-extractive socket was managed with obliterative suture only. In group 2, patients were treated with CaS placed into the sockets. Even if the occurrence of bleeding events was minimal, their frequency at day 1 postoperatively was significantly less in patients treated with CaS as compared with patients without. Bajkin et al. [32] also compared different local hemostatic solutions: (1) suture, (2) gelatin sponge into the socket without suturing and (3) neither of those two measures. Bleeding events in group 1, 2 and 3 were observed. All cases of bleeding were easily solved with local hemostatic measures. Halfpenny et al. [33] compared the use of a resorbable oxycellulose dressing with a fibrin adhesive. No discernible differences in terms of bleeding in the postoperative period was noted. Conversely, postoperative pain was reported more frequently in the group that used a resorbable oxycellulose dressing. TXA was also evaluated by Carter et al. [35], who compared it with an autologous fibrin glue preparation. Subjects in Group A were required to rinse with 10 mL of a 4.8% TXA solution 4 times a day for 7 days postoperatively, while patients in Group B received autologous fibrin glue intraoperatively. Overall, two patients from the autologous fibrin glue group presented postoperative bleeding and were found to have grossly elevated INR values on the day of bleeding. A second study by Carter et al. [36] proved the efficacy of the TXA, focusing on the mouthwash regimen to adopt in the postoperative days. Group A received postoperatively a 4.8% TXA mouthwash for a 2-day period. Group B received the same mouthwash and instructions postoperatively, to be continued for 5 days. Two patients in group A and one in group B had minor postoperative bleeds that required minor ambulatory intervention, but the difference was not statistically significant. Finally, Bacci et al. [39], in a large multicenter study, compared 451 patients in therapy with warfarin with 449 non-anticoagulated patients. The hemostatic measures used to control bleeding in the warfarin group were fibrin sponges, silk sutures and gauzes saturated with TXA. In the OAT group, seven events were observed, which were handled easily with local measures. In the control group, four cases occurred; however, the difference was not statistically significant.

#### 3.3.2. NOACs

Cocero et al. [41] conducted a retrospective study considering 100 patients treated with direct oral anticoagulants. They divided the patients into two groups: 64 patients with comorbidities (diabetes, liver disease, renal insufficiency) and 36 without comorbidities. Four bleeding episodes were observed in the comorbidities group and none in the non-comorbidity group. In the comorbidities group, the factors triggering the episodes were the extraction of more than one tooth and the proximity of extracted teeth. The last dose of DOAC has been taken at least 4 h before the intervention.

Kwak et al. [13], in a retrospective study that included 120 patients (153 dental extractions), analyzed bleeding episodes in different procedures that were divided in two groups depending on the risk assessment: high and low bleeding risk. Low bleeding risk procedures consisted of impression-taking, crown preparation, root canal therapy and resin filling. High bleeding risk procedures were simple and complex tooth extractions, scaling and implant surgery. Bleeding occurred in nine out of 153 cases: two cases of scaling, three cases of simple extraction, three cases of first stage implant surgery and one case of resin filling. The number of cases was higher for no discontinuance of NOAC therapy or 1 day of discontinuance than for 2 or 3 days of discontinuance.

Miller et al. [42] conducted a retrospective evaluation involving patients who underwent a total of 98 dental extractions. Overall, 42 teeth were extracted with rivaroxaban, 28 with Edoxaban, 22 with apixaban and six with dabigatran. Single and multiple extractions were performed on patients without bleeding complications regardless continuation or discontinuation of the DOAC.

Patel et al. [44] conducted a retrospective study, in which 82 patients underwent 111 dental extractions. In 35 procedures, advice was given to omit the DOAC, either before or after treatment. There was no bleeding following the majority of procedures. Persistent bleeding followed 15 procedures, of which seven procedures required specific intervention such as re-suture.

Miclotte et al. [45] conducted a prospective case–control study in 26 patients undergoing dental extraction and treated with dabigatran, rivaroxaban or apixaban, and 26 matched controls not taking any antithrombotic drug. Patients were instructed to skip only the dose on the morning of the procedure. A procedural bleeding score was recorded, and early and delayed bleeding was assessed post-operatively at days 1 and 7. There was no statistical difference in the procedural bleeding score or in early bleeding events (5 in both groups). However, delayed bleeding occurred more frequently in anticoagulated compared to non-anticoagulated patients.

Hanken et al. [46] focused on the risk of bleeding in patients taking Rivaroxaban, the most widely used novel oral anticoagulant. It was a retrospective cohort study that included 52 oral procedures performed under continued oral anticoagulant therapy with rivaroxaban 20 mg/day. Among them, two procedures were performed under continued dual therapy with aspirin 100 mg/day added to the regimen. Postoperative bleeding events were compared with 285 oral procedures in patients without any anticoagulation/antiplatelet therapy. It resulted that there were more episodes of bleeding in the study group under rivaroxaban therapy (15%) than in the control group (0.7%). All the episodes occurred within the first week and were manageable with local hemostatic measures such as gauze compression and fibrin sponges.

#### 3.3.3. VKAs and NOACs

In this group, the included studies considered patients in therapy with both VKAs and NOACs. The bleeding risk was compared together with the various local measures to control bleeding.

1. Implant Therapy

The study by Rubino et al. [47] actually did not consider patients undergoing tooth extractions, however the results deriving from implant and periodontal procedures have been reviewed as well. The authors considered patients under therapy with warfarin or novel anticoagulant, combined or not with antiplatelet drugs. Out of 867 procedures, the incidence of bleeding episodes was very low, being only three. The anticoagulant or antiplatelet therapy were not discontinued in any case, and the incidence of bleeding was 0.35%.

Clemm et al. [50] also performed implant-related treatments including single or multiple implant insertions, implant exposures, sinus floor augmentation and vertical and/or lateral bone grafting with autologous bone grafts. Subjects in the test groups were treated with platelet aggregation inhibitors (PAIs), Vitamin K inhibitors, Vitamin K inhibitor withdrawal bridged with LMWH or NOACs. Patients in the control group were non-anticoagulated. There were seven postoperative bleedings in 564 patients, four in the test group, and three in the control group. In the NOAC group, no episodes were observed. All bleeding events were easily controlled with local hemostatic measures.

2. Dental Extractions

Yoshikawa et al. [48] conducted a prospective observational study that included 367 patients undergoing tooth extraction, 119 receiving NOACs and 248 receiving warfarin. All extractions in NOAC patients were performed 6–7 h after therapy in consideration of the blood half-life under continued antithrombotic treatment. Postoperative bleeding episodes occurred in four cases in the NOAC group and in 23 cases in the warfarin group. However, note that in the warfarin group, more complex surgical extractions were performed. Therefore, the difference in terms of bleeding events was not considered statistically significant. All cases of postoperative bleeding in the warfarin group were managed by local hemostatic measures such as compression with gauze, without stopping the anticoagulant therapy. A total of four bleeding episodes were recorded in the NOAC group: two were managed with local measures, while the other cases required the discontinuation of NOAC for one dose, in order to obtain a standard level of coagulation.

Lababidi et al. [43] conducted a retrospective controlled cohort study. All patients underwent dental extractions and three groups were identified: 29 patients on chronic NOAC therapy, 14 patients under NOAC therapy who discontinued the drug and a control group of 50 patients on chronic warfarin therapy without peri-procedural cessation. The incidence, severity and timing of bleeding events were recorded for each group. In the 53 procedures conducted within the NOAC group, 15 were done following perioperative cessation of the medication under physician advice, with large variability in the timing of suspension, ranging from 1 to 14 days. Four bleeding events were reported in the non-ceased NOAC group, two of them requiring reintervention. No bleeding events were reported in the ceased NOAC group. Within the warfarin group, there were nine episodes, with five requiring reintervention. There was no statistically significant difference between the three groups.

Caliskan et al. [49], in a randomized controlled trial, considered four different groups: patients taking direct thrombin inhibitors, patients taking factor Xa inhibitors, patients taking warfarin and a drug-free control group. A total of 84 patients underwent simple tooth extractions. The number of patients showing mild and moderate bleeding was significantly higher in warfarin group compared to other groups, and also the amount of bleeding in warfarin group was apparently higher.

Mauprivez et al. [38], in a prospective observational study, examined 51 patients who were treated with oral anticoagulants and required dental extractions. They were divided into two groups: 31 patients receiving NOAC and 20 control patients taking VKA with an INR between 2.0 and 3.0. In both groups, extractions were performed under continued OAT, and the same local hemostatic measures were applied. In general, five patients taking NOACs showed seven bleeding episodes, whereas four patients treated with VKAs had five bleeding episodes during the postoperative follow-up period. The difference in the number of bleeding events between the two groups was not statistically significant. Only one episode required revision of the wound, application of fibrin glue and re-suturing.

Miranda et al. [51] performed a randomized clinical trial dividing 50 patients in two groups. Group A consisted of 12 patients treated with NOACs, while Group B consisted of 38 patients treated with Warfarin bridged with LWMH, due to an INR value > 3. The NOAC was not interrupted but the appointments were scheduled after 12 or 24 h from the last dose, so that the surgical procedures were performed with the lowest plasmatic concentration of the drug. Overall, 27% cases in Group B showed an increased intraoperative bleeding, resulting in a reduction of the visibility of the operative field and higher difficulty in surgical procedures. This event did not occur in Group A. In 15.78% of cases in Group B, widespread bleeding episodes required re-intervention, consisting in changing the systemic therapy with heparin and applying additional sutures at the surgical site. In Group A, a good management of the hemostasis was obtained, without intra- and post-operative bleeding complications.

Yagyuu et al. [52], in a recent retrospective cohort study, evaluated the primary outcome of bleeding after extractions. A total of 1196 tooth extractions in 541 patients fulfilled the inclusion criteria, with 72 extractions involving NOACs, 100 extractions involving VKAs and 1024 extractions involving controls with no anticoagulants. The incidences of postextractive bleeding per tooth for the NOAC, VKA and no anticoagulant extractions were 10.4%, 12.0% and 0.9%, respectively.

## 4. Discussion

In oral surgery, excessive bleeding is a negative factor that may compromise the surgical procedure itself and increase the post-operative morbidity and discomfort for the patient. In this regard, a strict preoperative evaluation of the bleeding risk and clear protocols on how to handle bleeding events may contribute to minimize the complications experienced by both professionals and patients.

The aim of the present review was to explore the existing literature related to the management of patients in therapy with anticoagulant drugs undergoing dental surgery. Most of the studies that we considered evaluated patients scheduled for dental extractions (one of the most daily and standard procedure), while only a few considered other procedures such as implant placement (single or multiple), implant exposures, sinus floor augmentation and bone grafting with autologous bone. The primary outcome was the evaluation of the post-operative bleeding. Additional parameters were also investigated, including the suspension of the anticoagulant regimen, the type of anticoagulant therapy and the local measures adopted to contain bleeding. A scoping review might be considered the best method of research for this topic due to the fact that we are not answering a specific question (like the systematic review approach), but we are trying to summarize a much broader field of interrogatives [15]. Three main typologies of studies were identified: studies including patients in warfarin therapy, studies treating patients in NOAC therapy and studies that compared the two categories.

The approaches commonly adopted in case of anticoagulated patients during dental extractions varied from total discontinuation of OAT to the continuation of therapeutic levels of anticoagulation. Increasing numbers of authors have stated that dental extractions in anticoagulated patients can be successful without altering the therapy regimen, laying emphasis on local hemostatic measures [53]. The constant monitoring of INR values in these patients is crucial in the pre-surgical planning. On the basis of the individual response of each patient, the accidental drop of INR values to subtherapeutic levels can often occur after discontinuation of OAT, and this could represent a risk for patients without additional prophylaxis [5,23].

The studies that suspended warfarin therapy aimed to obtain an INR level below 2.0 on the day of the intervention. There is no agreement between clinicians about the suspension regimen [4,20,23]. Bringing the INR values to the target level could result in an increased risk of severe consequences such as thromboembolic events that outweighs the risk of bleeding episodes during the extraction, as these episodes can be easily managed with local measures [27]. An important conclusion that can be drawn is that dental surgery can be safely conducted if INR levels are maintained between 1 and 4, controlling bleeding with the aid of local hemostatic agents.

As resulted in the present review, studies reporting on VKAs still represent the majority of literature with respect to anticoagulant drugs in dental patients.

Given the uncertainty about the ideal timing of drug suspension, an alternative strategy was often represented by bridging with LMWH. VKAs have a 40 h half-life. Thus, almost two days with a minimal blood concentration of drug may be dangerous and lead to severe thromboembolic consequences. It was therefore believed that a periprocedural heparin bridging strategy compared with no bridging would minimize the period of time without anticoagulation and thus reduce thromboembolic events at the expense of an acceptable increase in bleeding rates. This conceptual framework led to the generation of explicit protocols for periprocedural heparin bridging [54]. In this context, heparin bridging may be considered not useful anymore, because the fast onset time of the new anticoagulants brings the non-anticoagulation period from 2–3 days to much more manageable few hours.

Many studies considered the continuation of warfarin without cessation nor bridging. As different studies showed no differences between discontinuing and non-discontinuing warfarin in terms of bleeding events, the best solution is to continue anticoagulant therapy with VKAs. Once it is clear that non-discontinuation of warfarin may be considered the best approach, local hemostatic measures become absolutely necessary to control intra-surgical and post-operative bleeding.

Novel oral anticoagulants have proven to be safe and effective, offering a series of advantages including rapid action, no need for constant monitoring, few drug and food interactions, and a broad therapeutic margin. With such pharmacological advantages, it is not surprising that the number of such patients on NOACs being addressed by dental practitioners is increasing at a fast rate. Although in the present review there are still more studies on VKAs than NOACs, in the next few years the trend will probably reverse.

In 2018, Fortier et al. [55] reviewed the literature and concluded that, with the exception of a new reversal agent to direct thrombin inhibitors such as dabigatran, the lack of a specific antidote for factor Xa inhibitors makes the establishment of management protocols very difficult. Thus, caution should be taken when treating patients in therapy with NOACs until precise guidelines will be made available.

The recommended interruption of NOAC administration varies depending on the class of anticoagulant. The dental clinician should therefore become familiar with each of these time frames to inform the patient. Note that, if NOAC administration is interrupted pre-operatively, then NOAC re-administration should begin post-operatively following hemostasis [55]. In this matter, the discontinuation of NOACs was not an important factor in determining bleeding events in the studies considered in the present review [13,42,44].

A relevant advantage of these molecules is the possibility to plan the intervention based on the last dose of NOAC taken, in order to minimize the intraoperative bleeding without altering the anticoagulant regimen. Different studies adopted this method, so that the procedure was scheduled several hours from the latest dose or just before the subsequent dose of NOAC. Interestingly, in each study assessed in the present review, this approach resulted in an excellent view of the surgical field [41,48,51]. Although this may be considered a valid approach, there is no conclusive evidence that recommends scheduling the appointment based on the last dose of NOAC. In this respect, Brennan et al. recently suggested that there is no need to adjust DOAC dosing prior to dental extractions, nor is there a need to time the dental extractions around DOAC doses [56].

Many different hemostatic methods have been proposed and used over the last decades. In the present review various local measures were identified, such as compression with gauze moistened with saline solution, dry gauze compression, TXA in the form of moistened gauzes and postoperative mouthwash, fibrin and gelatin sponges, autologous fibrin glue, resorbable oxycellulose dressing, N-butyl-2-cyanoacrilate glue (NBCA), epsilon-aminocaproic acid (EACA) and calcium sulfate (CaS) amongst others. Suture is not necessary if the socket is well represented in terms of well-represented bone walls and restraining defect. Note in this matter that some authors observed more bleeding events in sutured alveoli rather than in alveolar sockets left to heal spontaneously [23].

EACA affects the fibrinolytic system at several points. It acts primarily as an inhibitor of plasminogen activation, and only at high concentration (5 × l0^2M) is able to inhibit plasmin directly. EACA is not as effective and safe as the TXA, which is 10 times more potent, but its action can be compared with common local measures such as gauze compression and fibrin sponges. [26] In fact, in the study conducted by Da Silva et al. [25], out of 140 patients, 70 in the study group using EACA and 70 in the control group using local pressure and other local measures, the authors reported a comparable incidence of late bleeding between the two groups, being 15.7% and 17.1%, respectively. Considerable uncertainty thus remains about the efficacy and safety of EACA.

CaS has been widely employed throughout the years in different applications. It has been used in bone regeneration as a graft material and graft binder/extender and as a barrier in guided tissue regeneration. It has been shown that tissue often migrates over CaS if primary closure cannot be achieved [57]. Scarano et al. [31] exploited this aspect by placing CaS into the post-extractive sockets. Bleeding frequency at the first postoperative day was significantly lower in patients treated with CaS compared to patients CaS-free. No statistically significance differences in bleeding were noted after some days, meaning that CaS could be used to obtain a better hemostasis in the early stages of healing.

NBCA-based tissue adhesive was employed as a non-suture method to seal wounds in oral surgery. Al Belasy et al. [24] and previous studies [58] confirmed the efficacy of this local hemostatic. Al Belasy compared patients treated with Hystoacril glue placed in the socket right after the extraction (three different layers, allowing 30 s of time between applications) with patients treated with ordinary local measures such as gelatine sponges. Both the groups obtained an adequate hemostasis after the extractions, but it is remarkable how in the NBCA group the hemostasis was immediate, while it took between 10 and 20 min in the ordinary group. NBCA proved its efficacy also in terms of late bleeding, as no episodes occurred in this group in contrast to the five late episodes encountered in the ordinary group.

The most widely used hemostatic protocol to date is compression and mouthwash rinses with TXA [20,21,25,27,33,35,36,39]. The different formulations of TXA do not seem to affect its effectiveness. Indeed, although mouthwash is the most reported formulation, it may also be used in dressings with gauze, for local irrigation (250 mg/5 mL solution), as a suspension of a tablet (250 mg) crushed with saline or local anesthetics and used in gauze or even directly on the wound. TXA is a synthetic derivative of the amino acid lysine and exerts its antifibrinolytic effect through reversible blockade of lysine binding sites on plasminogen molecules. Although plasmin can still be formed under these circumstances, it is unable to bind to and degrade fibrin. [59] In the present review, the most prescribed concentration of TXA mouthwash was 4.8% and the postoperative regimen ranged from 2 to 5 up to 7 days. All these formulations resulted effective in containing bleeding episodes.

Even if no specific studies have been found in this respect, almost all authors use gauze compression as the first hemostatic measure. Moreover, TXA has been widely studied indirectly as it is often used as a reference local measure in control groups in the comparison with other hemostatic agents. Hemostasis by compression was obtained in different modalities: the gauze can be dry, it can be moistened with saline solution or it can be moistened with hemostatic agents like TXA. It is the authors’ opinion, based on the everyday clinical experience, that using dry gauze can dislodge the blood clot, so it is advisable to always wet these devices. In a study, the time required to attain hemostasis was higher when using gauzes moistened with saline compression compared to TXA, and this difference was statistically significant. However, despite the slightly delayed effect in comparison with TXA, compression with gauzes appears to be a safe method in controlling postoperative bleeding in ambulatory dental surgery, particularly when used preventively in patients taking oral anticoagulants and also when used as secondary measures for the control of delayed bleeding [21].

## 5. Conclusions

Anticoagulant therapy with warfarin, whether continued, stopped or bridged a few days before surgery with heparin therapy, seems to be no longer preferred in clinical settings. This is mainly related of the difficult management of vitamin K antagonists, their interaction with numerous drugs and foods and their constant need to be monitored for effectiveness and blood concentration. The trend is now directed toward the prescription of direct oral anticoagulants, which have far fewer interactions and better perioperative management. The four new oral anticoagulants, namely dabigatran, rivaroxaban, apixaban and edoxaban evaluated in the present review showed effectiveness and safety, despite some differences in indications by to age groups. In case of extractive surgery, one of the most widely adopted protocols to reduce the risk of bleeding is to perform surgery as far away as possible from the last administration, with no drug suspension. All bleeding episodes that occurred in the studies reviewed herein were easily controlled using conventional hemostasis techniques. Many different hemostatic agents have been investigated, including fibrin and gelatin sponges, autologous fibrin glue, resorbable oxycellulose dressing, N-butyl-2-cyanoacrylate glue, epsilon-aminocaproic acid, calcium sulphate and suturing. The most effective, reliable and widely used in the literature is apparently compression with gauze moistened with saline solution or tranexamic acid, which can also be used effectively in the form of mouthwashes. As literature is extremely lacking in providing evidence on the management of new oral anticoagulants in dental surgery, further clinical studies are needed to confirm these initial conclusions.

## Figures and Tables

**Table 1:** Studies considering VKAs.

Author	Description of Primary Outcome	Anticoagulant	Sospension of Anticoagulant	Other Drugs	Type of Oral Surgery Procedure	Monitoring Time and Follow Up	Method of Bleeding Control	Number of Partecipants	Number and Type of Bleeding Events
B. Bajkin, I. Bajkin, B. Petrovic.	Lockhart definition of bleeding event: bleeding wich continues for more than 12 h; the patient returns to dental practice or emergency department; large hematoma or ecchymosis within the oral soft tissues; need of blood transfusion.	Acenocoumarol.	No.	Antibiotic; Painkiller, if necessary.	Teeth extraction.	2 h; follow up 5 days, with day 1 and day 2 controls.	Collagen sponge in extraction sockets; no suture; gauze pressure for 30’. In case of post-operative bleeding: gauze compression and eventually suture; administration of vitamin K, prothrombin complex concentrate or fresh frozen plasma.	213	Postoperative bleeding: 3 in group anticoagulant-aspirin; 2 in group only anticoagulant; no in aspirin group.
B. Bajkin, S. Popovic, S. Selakovic.	Hemorrhage: event requiring local pressure or additional surgical intervention. Immediate (first 2 h) or late.	Acenocoumarol; Warfarin	Yes; Stop 3–4 days before (INR of < 1.5); Bridging with heparin; Stop heparin 12 h before; Resumed on evening.	Antibiotic; Painkiller, if necessary.	Teeth extraction.	2 h after surgery; follow up 7 days, with day 1, day 2, day 4 controls.	Gauze compression for 30’; resorbable collagen sponges. In case of post-extraction bleeding, haemostatic agents and suture; vitamin K therapy.	214	Immediate bleeding (gauze compression): 8 in the continuation group; 5 in the stop group. second manifested post-extraction bleeding. No thromboembolic complications.
R. Sacco, M. Sacco, M. Carpenedo, P. Mannucci.	1- Mild bleeding: < 10’; 2-Moderate: 10–20’; 3-Severe: requiring a new surgical intervention or transfusion.	Acenocoumarol; Warfarin.	Yes, in control group: dose reduction (INR 1.8 mantained up to 24 h after surgery); full anticoagulant dosage resumed 48 h later. No, in test group (INR > 2.5).	Antibiotic; Painkiller, if necessary.	Teeth extractions (nontraumatic technique, flap elevation if necessary); Removal of cystic formations; Insertion of implants.	120’ after surgery; Follow up 6 days (contacted daily by phone); day 7, suture removal.	Control group: no local or general hemostatic measures; Test group: gelatin and oxidized cellulose sponges; tranexamic acid (local applications and mouthwashes, 4/day for 2 days). In case of bleeding: new suture; removal of clots; tranexamic acid mouthwashes.	131	No bleeding in the first 24 h; Mild postsurgical: 10 cases (15.1%) in control group and in 6 cases (9.2%) in the test group (9 warfarin and 7 acenocoumarol); No severe bleeding.
P. Cannon, V. Dharmar.		Warfarin (1.5–7.5 mg, mean 3.9 mg).	Yes, in control group; Resumed on the same day.	Antibiotic prophylaxis if necessary; Paracetamol if necessary; Avoid aspirin and nonsteroidal antiinflammatory drugs.	Teeth extraction (sometimes with flap elevation).	30’ after surgery; follow up 7 days with day 3 and day 5 controls.	Control group: local haemostatic agent Surgicel^®^ and 3/0 catgut suture (in study group only if involving bone or soft tissue). Gauze compression for 20’.	70	0 immediate post-operative bleeding during the 30’ after extraction; intermittent oozing in the first 24 h (3 in the control group and 2 in the study group). No serious bleeding or thromboembolic event.
S. Al-Mubarak, M. Rass, A. Alsuwyed, A. Alabulaaly, S. Ciancio.		Warfarin (2–10 mg).	Yes, in 2 group, Stop warfarin 2 days prior to surgery; Resume treatment 12 h after.	Antibiotic; Painkiller, if necessary.	Teeth extraction.	Follow up 7 days; phone contact in case of problems.		168	Percentage of bleeding in postoperative day 1: 12,9% (INR 1–2); 18.9% (INR 2–3); higher (INR>3). No intervention needed. No thromboembolic event.
A. Rocha, S. Oliveira, A. Souza, D. Travassos, L. Abreu, D. Ribeiro, T. Silva.	Storage of the fluids aspirated during the surgical procedure using a portable vacuum pump. Score 1: samples up to 5 mL; score 2: 6–10 mL; score 3: 11–15 mL, and so on. Post-operative bleeding and wound healing (satisfactory, swelling/erythema, or bone exposure).	Warfarin (5 mg).	No.	Antibiotic prophylaxis, if necessary; Paracetamol.	Teeth extraction.	60’ after surgery; Follow up 7 days; phone contact in case of problems.	3.0 nylon sutures; gauze compression for 20’. In case of immediate bleeding: absorbable gelatin sponge, tranexamic acid and/or new sutures.	138	Bleeding complications: 7 in anticoagulant group; 4 in control group. No post-operative late bleeds requiring hospitalization and/or blood transfusions (only haemostatic measures).
E. Soares, F. Costa, T. Bezerra, C. Nogueira, P. Silva, S. Batista, F. Sousa, C. Fonteles.	Postoperative hemorrhage: bleeding that could not be controlled by gauze compression during 20’, requiring medical intervention.	Warfarin (INR 2.1–3.1).	No.	Antibiotic prophilaxis, if necessary; Paracetamol;Avois aspirin and anti-inflammatory drugs for 10 days.	Teeth extraction (>2).	Follow up 7 days; phone contact by operator (12 h; 24 h); by the patients (if persisting bleeding even after 20 min of gauze compression, or pain, or fever).	If persistent bleeding: inspection and curettage of surgical site; fibrin sponge; X suture; 8’ compression with gauze soaked in 4.8% tranexamic acid.	65	Postsurgical bleeding 3.6–7.1% (mean of 5.3%) with tranexamic acid and fibrin sponge; 3.6% with gauze compression alone. In total 4 bleeding episodes
G.Sammartino, G. Marenzi, A. Miro, F. Ungaro, A. Nappi, J. Sammartino, F. Quaglia, C. Mortellaro.		Warfarin (INR 2-4); single/dual anticoagulation therapy.	Yes, in control group; Stop some days before (INR < 2); No, in test group (INR > 29.	Avoid cephalosporins, macrolides, quinolones (interfering with the coagulation; Diclofenac.	Teeth extraction (>2).	Follow up 7 days; phone contact in case of problems.		84	6 hemorrhagic complications (7.2%). 4 in the control group (late postoperative hemorrhage, 2–4 days after extraction period, excessive coagulum; 2 in the study group, immediate postoperative hemorrhage).
S. Queiroz, V. Silvestre, R. Soares, G. Campos, A. Germano, J. da Silva.	1- Absent bleeding (no complaints), 2- Little (bloodstained), 3-Moderate (some amount of blood in the mouth), 4-Severe (large amounts of blood in the mouth).	Warfarin (mean INR 2.4).	No.	Analgesic: dipirona or paracetamol. Antibiotic prophylaxis if necessary.	Teeth extraction (without flap elevation and ostectomy).	Follow up 7 days, with 12 h, 24 h, and 7 days controls.	Test group: gauze compression (5’); suture; Control group: compression (5’) with gauze soaked in tranexamic acid. For postoperative bleeding, the procedure was repeated.	37	Severe bleeding in 3 (8.1%) cases, mild in 19 (51.4%) and absent in 15 (40.5%) cases. In the first 12 h postoperatively, hemorrhage was moderate in 20 cases (54.1%), mild in 13 (35.1%), and absent in four (10.8%). On the seventh postoperative day, hemorrhage was absent in all cases; Time to achieve cessation of bleeding: 9.1 (±3.6) minutes; lower for the study group.
F. Al-Belasy, M. Amer.		Warfarin.	No.	Amoxicillin or Azithromycin; Paracetamol, if necessary; Avoid aspirin and other nonsteroidal antiinflammatory for 10 days after surgey.	Teeth extractions (also with alveolplasty and mucoperiostal flap).	Follow up 10 days; phone contact in case of problems.	Test group: histoacryl glue; interrupted resorbable sutures; Control and negative groups: gelatin sponge and multiple interrupted resorbable sutures.	40	5 in control group, postoperative spontaneous bleeding requiring treatment.
E. Karsli, Ö. Erdogan, E. Esen, E. Acartürk.	Intraoperative bleeding: weight of gauze swabs used before and after tamponade measured with a fine electronic weight measurement device; Postoperative bleeding: patients count the number of extra gauze swabs used for bleeding control during the first 48 h.	Warfarin.	Yes, in 2 groups; Stop 3 days before, then heparin bridging up to 24 h before (INR < 2); Resumed heparin after haemostasis and warfarin 48 h after (hospitalised); No, in test group.	Antibiotic; Painkiller, if necessary.	Teeth extraction.	20’ after surgery; follow up 7 days, with 48 h control.	Gauze compression; oxycellulose dressing; suture 3.0.; gauze compression for 1 h.	40	Higher values of Amount Of Bleeding in continuation warfarin group; Mean amounts of bleeding were 2.500 mg in continuation warfarin group; 1.000 mg in stop warfarin group; 1.288 mg in stop warafarin and unfractioned heparin; 1.736 mg in healthy group. No severe postoperative bleeding in any patient; number of used extra gauze swabs did not differ significantly among groups.
A. Scarano, B. Sinjari, G. Murmura, E. Mijiritsky, F. Iaculli, C. Mortellaro, S. Tetè.	Bleeding events measured by the scar tissue over the sockets: incomplete closure (poor healing); solid cloth over the socket (no bleeding); cloth that sheds and oozing tissue (positive bleeding).	Warfarin.	No.	Antibiotic; Painkiller, if necessary.	Teeth extraction.	1 h after surgery; follow up 7 days, with day 3, day 5 controls.	Control group: only suture; Test group: suture plus socket filled with CaS in layers.	30	No bleeding in suture group; some bleedig in CaS group on day 1.
B. Bajkin, S. Selakoviü, S. Mirkoviü, I. Šarþev, A. Tadiü, B. Milekiü.	Bleeding event if it is not handle by the patient by himself (gauze pressure). Immediate or delayed.	Warfarin.	No.	Antibiotic; Painkiller, if necessary.	Teeth extraction.	2 h after surgery; follow up 5 days, with day 1, day 3 controls.	Suture; absorbable gelatin sponge; gauze pressure.	90	Postoperative immediate bleeding: 1 (3.3%) in gelatine sponge group A; 2 (6.7%) in other groups.
W. Halfpenny, J. Fraser, D. Adlam.	Immediate or delayed. Severity scale of post-operative pain: no pain, moderate pain, severe pain.	Warfarin.	No.	Antibiotic; Painkiller, if necessary.	Teeth extraction.	Follow up 7 days; phone contact in case of problems.	Gauze compression; resorbable oxycellulose or fibrin adhesive; suture.	46	No immediate postoperative bleeding; 1 postextraction hemorrhage (24 h) re-sutured in oxycellulose group and 1 in fibrin group; 1 hospitalizasione for persistent intermittent bleeding in fibrin group; postoperative pain more frequent in oxycellulose group.
I. Evans, M. Sayers, A. Gibbons, G. Price, H. Snooks, A. Sugar.	Immediate or delayed. Sought help by phone is also a bleeding event.	Warfarin.	Yes, in control group; Stop 2 days before surgery; No, in test group.	Antibiotic; Painkiller, if necessary.	Teeth extraction.	10’ after surgery; Follow up 7 days; phone contact in case of problems.	Gauze compression for 10’; socket packed with oxycellulose dressing; suture.	109	Immediate postoperative bleeding: 3 in at and 3 in ctr; delayed postoperative bleeding 9 in at and 7 in ctr; rate of bleeding complications: 26% at and 14% ctr.
G. Carter, A. Goss, J. Lloyd, R. Tocchetti.	Event that can’t be controlled by biting the gauze pad for 20 min. Pain, edema, haematoma.	Warfarin.	No.	Antibiotic; Painkiller, if necessary.	Teeth extraction.	Follow up 7 days, with day 1, day 3 controls.	Test group: tranexamic acid rinse after extraction; absorbable oxidized cellulose mesh placed in the apical third; resorbable suture. Control group: Surgicel in the apical third; application of fibrin glue to the socket walls, suture and final application of the glue.	49	No bleeding complications in TxA group; 2 light bleedings on day 2 in fibrin glue group. Both required intervention.
G. Carter, A. Goss.	Immediate or delayed. Occurence of haematoma or oedema. Pain.	Warfarin.	No.	Antibiotic; Painkiller, if necessary.	Teeth extraction.	20’ after surgery; follow up 7 days, with day 1, day 3 controls.	Irrigation with 4.8% tranexamic acid mouthwash; oxycellulose sponge submerged in TA placed in the socket; resorbable suture; gauze compression for 20’.	85	Similar low bleeding rate; postoperative bleeding: 3 (severe periodontitis).
C. Bacci, M. Maglione, L. Favero, A. Perini, R. Di Lenarda, M. Berengo, E. Zanon.		Warfarin.	No (INR 1.8–4).	Antibiotic prophylaxis, if necessary; Paracetamol or Ibuprofen if necessary; Avoid aspirin.	Teeth extraction.	Follow up 8 days with day 3 control.	Fibrin sponges; silk sutures; gauze compression with tranexamic acid for 30’–40’; ice bag for 1 h.	898	Bleeding complications: 7 in anticoagulant group; 4 in control group; local haemostatic measures).
R. da Silva, T. Gadelha, R. Luiz, S. Torres.	1-No bleeding, 2-Mild (blood in the saliva), 3-Moderate (gauze compression), 4-Severe (surgical re-intervention and/or hospital admission).	Warfarin; with/not Antiplatelet agents.	No.	Antibiotic prophylaxis, if necessary.	Single tooth extraction.	20’ after surgery; follow up 7 days. 7 days postoperatively.	Bidigital alveolar compression with sterile gauze for 5’ min; intra-alveolar EACA in test group.	52	1 immediate bleeding in test group; 23 late bleeding (16.4%): 11 (15.7%) in test group and 12 (17.1%) in control group. Of these 23: 18 (78.3%) moderate(controlled by the patient by gauze pressure); 5(21.7%) required re-intervention.

**Table 2:** Studies considering NOACs.

Author	Description of Primary Outcome	Anticoagulant	Sospension of Anticoagulant	Other Drugs	Type of Oral Surgery Procedure	Monitoring Time and Follow Up	Method of Bleeding Control	Number of Partecipants	Number and Type of Bleeding Events
S. Miller, C. Miller.		NOAC, Rivaroxaban (42 teeth); Edoxaban (28); Apixaban (22); Dabigatran (6).	Yes, in 9/12 cases; Stop NOAC 52.5 h mean prior to surgery (12–120 h).	Antibiotic; Painkiller, if necessary.	Teeth extraction.	Follow up 15 days; phone contact in case of problems.		11.320	Bleeding complications were not reported for patients whose drug was discontinued or continued.
E. Kwak, S. Nam, K. Park, J. Huh, W. Park.		NOAC.	Yes; Stop on the day (50); 1 day before (37); 2 days (23); longer (10).	Antibiotic; Painkiller, if necessary.	Restaurative dental treatments; Curettage; Simple (38) or surgical (13) tooth extractions; Implant surgery.	Follow up 7 days; phone contact in case of problems.	Hemostatic agents (absorbable collagen sponge; oxidized regenerated cellulose). For mucoperiosteal flap, 3–0 or 4–0 suture. Gauze compression for 1 h.	120	Postoperative bleeding: 9/153 (2 scaling, 3 simple extraction, 3 implant surgery, 1 resin filling); 24–72 h after; Higher in no discontinuance or 1 day of discontinuance group.
J. Patel, S. Woolcombe, R. Patel, O. Obisesan, L. Roberts, C. Bryant, R. Arya.	1-bleeding present, no action; 2-consultation in the dental unit, no intervention; 3-surgical intervention (resuturing and haemostatic packing) and/or antifibrinolytic; 4-blood transfusion, replacement therapy or desmopressin.	NOAC.	Yes, minimise at the time of surgery; Stop before and/or after surgery.	Antibiotic; Painkiller, if necessary.	Teeth extraction.	30’-60’ after surgery; Follow up 7 days; phone contact in case of problems.	Gauze compression with 5% tranexamic acid for 30’–60’; haemostatic packing; suture.	82	Persistent bleeding: 15 (7 required specific intervention).
N. Cocero, M. Basso, S. Grosso, S. Carossa.	1-Mild bleeding: oozing; 2-Moderate: not manageable by the patient; 3-Severe: not manageable with topical hemostatic measures (systemic therapy and/or hospitalization).	NOAC: Dabigatran; Apixaban; Rivaroxaban.	Yes, Stop NOAC 4 h before surgery.	Antibiotic prophylaxis, if necessary; Paracetamol.	Teeth extractions (<3; atraumatic manner, no mucoperiosteal flap raised and without rotary instruments).	Follow up 7 days, with day 1, day 3 controls.	Digital mechanical pressure and topical agents (resorbable gelatin sponges). For wide alveolar sockets, sutures 3-0 silk; dressings with 5% tranexamic acid. Moderate bleeding: reintervention with removal of necrotic clot and new suture (suspension of the NOAC until following morning).	100	4 bleeding episodes (1 moderate 1 h after and 3 mild on day 1) in comorbidity group; None in the non-comorbidity group; Overall bleeding rate, 4 of 100 (4%); 0/36 (0%; without comorbidities); 4/64 (6.25%; with comorbidities).
H. Hanken, A. Gröbe, M. Heiland, R. Smeets, L. Kluwe, J. Wikner, R. Koehnke, A. Al-Dam, W. Eichhorn.		Rivaroxaban (20 mg/day) plus/not aspirin (100 mg/day).	No.	Antibiotic prophylaxis, if necessary; Ibuprofen for 3 days.	Teeth extraction.	Follow up 7 days; phone contact in case of problems.	Collagen fleece; suture. In case of bleeding: compression; fibrin glue; new suture.		All bleeding events in the first week; more bleeding complications in patients taking rivaroxaban (11.5% vs. 0.7%).
I. Miclotte, M. Vanhaverbeke, J. Agbaje, P.Legrand, T. Vanassche, P. Verhamme, C. Politis.	Amount of bleeding during the extraction, scored on a scale from 1 to 5 (1:no bleeding; 5: continued bleeding despite standard measures). Early (day 1) and delayed (day 7) bleeding.	Rivaroxaban (69%); Dabigatran; Apixaban.	Yes; Skip only the dose on the morning; Resumed 4 h after.	Antibiotic; Painkiller, if necessary.	Teeth extractions (mean 2.6; syndesmotome followed by forceps extraction with/without osteotomy).	Follow up 7 days, with day 1 control.	Suture with Vicryl^®^ 3-0.	52	No difference in the procedural bleeding score (3.15 in the NOAC group versus 2.92 in the control group); Early bleeding events: 5 in both groups; Delayed bleeding: 7 in anticoagulated group, 0 in non-anticoagulated group. 12 patients in the NOAC group and 5 patients in control group had a bleeding event; No major bleeding events.

**Table 3:** Studies considering both VKAs and NOACs.

Author	Description of Primary Outcome	Anticoagulant	Sospension of Anticoagulant	Other Drugs	Type of Oral Surgery Procedure	Monitoring Time and Follow Up	Method of Bleeding Control	Number of Partecipants	Number and Type of Bleeding Events
R. Rubino, R. Dawson, R. Kryscio, M. Al-Sabbagh, C. Miller.		Dabigatran; Rivaroxaban; Apixaban; Edoxaban; Warfarin; (other antiplatelet drugs).	No, in 99.6% of patients; Yes, in 4 patients (1–5 days).	Antibiotic; Painkiller, if necessary.	Scaling and root planing (484); implant placements (218); Open flap debridements (23); Gingival grafts (16); Sinus lift with lateral window (15); Other (71).	Follow up 7 days; phone contact in case of problems.		456	Postoperative bleeding: 3 (0.35%); resolved with local hemostatic measures.
M. Miranda, L. Martinez, R. Franco, V. Forte, A. Barlattani, P. Bollero.	(1)extra-alveolar clots and bleeding after more 24 h, requiring reoperation; (2) bleeding < 24 h, controlled by gauze pressure; (3) uncontrollable bleeding after 24 h, requiring reoperation; (4) haematomas; pain.	Dabigatran; Rivaroxaban; Apixaban; Warfarin.	Yes, in group 2 (INR > 3); Stop warfarin Bridging with heparin; Stop heparin 12 h before; Resumed 1 day after. No, in NOAC group.	Antibiotic; Painkiller, if necessary.	Teeth extraction.	60’ after surgery; follow up 7 days, with day 1, 3 day 3 controls.	Socket irrigation with tranexamic acid; gelatine sponge; suture; gauze compression with tranexamic acid for 15’; mouth rinses with a 10 mL of 5% tranexamic acid solution for 2’, 4/day, for 7 days.	50	Postoperative bleeding: 12 patients in warfarin group; no episodes NOAC group.
H. Yoshikawa, M. Yoshida, M. Yasaka, H. Yoshida, Y. Murasato, D. Fukunaga, A. Shintani, Y. Okada.	Oozing or marked haemorrhage that could not be stopped by gauze compression, requiring medical intervention.	NOAC (119: 32 dabigatran, 31 rivaroxaban, 39 apixaban, 17 edoxabaN); Warfarin (248).	No, extractions 6–7 h after last dose; Resumed after haemostasis.	Antibiotic; Painkiller, if necessary.	Teeth extraction: nonsurgical extraction; surgical extraction (mucoperiosteal flap and/or osteotomy); impacted tooth extraction.	Follow up 7 days; phone contact in case of problems.	Resorbable gelatin sponge; 3-0 silk sutures; gauze compression for 1 h.	367	Postoperative bleeding: 4 (3.1%) in NOAC group; 23 (8.8%) in warfarin group.
M. Caliskan, H. Tükel, M. Benlidayi, A. Deniz.	Intra-operative bleeding measured with Karsli-Erdogan method: avoid contaminations to the blood isolating salivary ducts; gauze swabs used and then weighted.	NOAC; Warfarin.	No.	Antibiotic; Painkiller, if necessary.	Teeth extraction.	20’ after surgery; follow up 7 days, with day 2 control.	Gauze compression for 20 min; oxidized cellulose dressing; suture 3.0.	86	The mean AOB in warfarin group was significantly higher than the other groups (1.388 mg; 1.909 mg; 3.673 mg; 1.593 mg); mild bleeding on day 2: 1, 2, 6, 0; moderate bleeding on day 2: 1, 2, 6, 0; mild bleeding on day 7: 0, 1, 1, 0; moderate bleeding on day 7: 0, 0, 2, 0; none hospitalization.
E. Lababidi, O. Breik, J. Savage, H. Engelbrecht, R. Kumar, C. Crossley.		NOACs, warfarin	No.	Antibiotic; Painkiller, if necessary.	Teeth extraction.	Follow up 7 days; phone contact in case of problems.	Haemostatic agent; suture. Delayed bleeding: re-packing of haemostatic agent, gauze pressure and temporary cessation of rivaroxaban.	93	Minor bleeding events: 4 (10.5%) in continuation NOAC group; 9 (15.3%) in warfarin group. No bleeding in stop NOAC group.
C. Mauprivez, R. Khonsari, O. Razouk, P. Goudot, P. Lesclous, V. Descroix.	Persistent oozing or marked hemorrhage over 20 min after tooth extraction despite local hemostasis; bleeding episode during the first 7 days.	VKAs and NOACs	No, in the test group; Yes, in the control group.	Antibiotic; Painkiller, if necessary.	Teeth extraction.	30’-40’ after surgery; follow up 8 days, with day 3 control.	Oxidised cellulose; resorbable suture; gauze compression with tranexamic acid for 30’–40’; ice bag for 1h.	900	Bleeding events: 7 in anticoagulant group (6/7 late, 2 days after; 1/7 6 days after); 4 in the control group (2 days after); managed with surgical treatment and suture.
R. Clemm, F. Neukam, B. Rusche, A. Bauersachs, S. Musazada, C. Schmitt.	Intraoperative bleeding: low, moderate, severe; early bleeding (up to 24 h after); late bleeding (>24 h).	Warfarin; Heparin (8 bridging); NOAC: Dabigatran (6); Rivaroxaban (8); Apixaban (2); Antplatelet (63, 21 plus anticoagulation).	Yes, in 8 patients; heparin birdging.	Antibiotic in bone augmentationand/or an implant insertion or for antibiotic prophilaxis.	Dental implants; Sinus floor augmentation; Vertical and/or lateral bonegrafting with autologous bone grafts.	Follow up 10 days, with day 1 control.	Suture Vicryl 5.0; in case of bleeding: gauze compression with tranexamic acid; additional suture; revision.	564	7 postoperative bleedings in 564 patients (1.2%). 4 in anticoagulant groups(3.4%), 3 in the non-AT group (0.6%). No thromboembolic complication. 2 hospitalized (1 in platelet aggregator inhibitor group; 1 in non-AT group). No postoperative bleeding in NOAC group.
T. Yagyuu, M. Kawakami, Y. Ueyama, M. Imada, M. Kurihara, Y. Matsusue, Y. Imai, K. Yamamoto, T. Kirita.	Bleeding that could not be stopped by gauze pressure, requiring medical treatment between 30min and 7 days after.	Warfarin; NOAC.	No.	Antibiotic; Painkiller, if necessary.	Teeth extraction.	30’ after surgery; follow up 7 days.	Oxidised cellulose or gelatine sponge; suture.	543	Postextraction bleeding: 10.4% NOAC; 12% Warfarin; 0.9% no anticoagulant; local hemostatic measures.

## Abbreviations

PT (prothrombin time), INR (international normalized ratio), VKAs (vitamin K antagonists), NOAC (new oral anticoagulants), DOAC (direct oral anticoagulants), NBCA (N-butyl-2-cyanoacrilate), CaS (calcium sulfate), EACA (epsilon amino caproic acid), TXA (tranexamic acid).
